# Systematic Review of Acupuncture for Chronic Prostatitis/Chronic Pelvic Pain Syndrome

**DOI:** 10.1097/MD.0000000000003095

**Published:** 2016-03-18

**Authors:** Zongshi Qin, Jiani Wu, Jing Zhou, Zhishun Liu

**Affiliations:** From the Department of Acupuncture (ZQ, JW, JZ, ZL), Guang’anmen Hospital, China Academy of Chinese Medical Sciences; and Beijing University of Chinese Medicine (ZQ, JZ), Beijing, China.

## Abstract

Acupuncture is a promising therapy for relieving symptoms in chronic prostatitis/chronic pelvic pain syndrome (CP/CPPS), which affects >15% of adult men worldwide.

The aim of the study was to assess the effects and safety of the use of acupuncture for CP/CPPS.

MEDLINE, EMBASE, CENTRAL, Web of Science, CBM, CNKI, Wang-Fang Database, JCRM, and CiNii were searched from their inception through 30 November 2015. Grey literature databases and websites were also searched. No language limits were applied.

Only randomized controlled trials (RCTs) with CP/CPPS treated by acupuncture were included. Two reviewers extracted data and assessed the risk of bias of RCTs using the Cochrane Risk of Bias Tools, respectively.

Seven trials were included, involving 471 participants. The result of meta-analysis indicated that compared with sham acupuncture (MD: −6.09 [95%CI: −8.12 to −5.68]) and medicine (Levofloxacinand, Ibuprofen, and Tamsulosin) (MD: −4.57 [95%CI: −7.58 to −1.56]), acupuncture was more effective at decreasing the total NIH-CPSI score. Real acupuncture was superior to sham acupuncture in improving symptoms (pain, voiding) and quality of life (Qof) domain subscores. Compared to sham acupuncture and medicine, acupuncture appears to be more effective at improving the global assessment. Two trials found that there is no significant difference between acupuncture and sham acupuncture in decreasing the IPSS score. Acupuncture failed to show more favorable effects in improving both symptoms and the Qof domain compared with medicine.

Overall, current evidence supports acupuncture as an effective treatment for CP/CPPS-induced symptoms, particularly in relieving pain. Based on the meta-analysis, acupuncture is superior to sham acupuncture in improving symptoms and Qof. Acupuncture might be similar to medicine (Levofloxacinand, Ibuprofen, and Tamsulosin) in its long-term effects, but evidence was limited due to high ROB among included trials as well as potential heterogeneity. Acupuncture is associated with rare and slightly adverse events.

**Protocol registration** PROSPERO CRD42015027522.

## Abstract 2

Supplemental Digital Content is available in the text

## INTRODUCTION

Chronic prostatitis/chronic pelvic pain syndrome (CP/CPPS) is 1 of 4 different categories of chronic prostatitis classified by the National Institute of Health (NIH) and is different from the first and second types. CP/CPPS primarily presents with pain symptoms in the prostate region in the absence of any urinary tract infection for at least 3 months of a 6-month period.^1^ Aside from pain symptoms, CP/CPPS is often associated with negative cognitive, behavioral, sexual or emotional consequences, as well as with symptoms suggestive of lower urinary tract and sexual dysfunction, and affects adult men worldwide.^2–4^

Based on a survey in China, the prevalence of CP/CPPS-like symptoms among Chinese men is 4.5%.^5^ Moreover, it is estimated that CP/CPPS comprises 90% of the prostatitis syndromes among patients.^6^

Pharmacological interventions are often prescribed for patients with CP/CPPS. Drugs commonly prescribed for CP/CPPS management include antibiotics, anti-inflammatories (NSAIDs), alpha-adrenergic blockers, and neuromodulatory drugs.^7–11^ Given its complex etiology and pathogenesis, CP/CPPS is still one of the least understood diseases in urology and can present a major challenge to health care providers due to its poor response to therapy. In 2004, Nickel et al found that it may be problematic to treat CP/CPPS with monotherapeutic strategies; multimodal treatment aimed at primary symptoms is necessary, and comorbidity must be taken into consideration.^10,12,13^

As a form of complementary treatment, acupuncture has been performed on patients with urinary diseases in Eastern nations for a long time but has been limited by the insufficient number of high-quality, well-designed randomized controlled trials (RCTs).^14^ The evidence level of evidence-based medicine is still low.^1^ In 2012, Posadzki et al conducted a systematic review focused on acupuncture for CP/CPPS^15^. According to this review, acupuncture's treatment of CP/CPPS is encouraging. However, as previously highlighted, the quantity and quality of trials included in the review hindered the researchers’ ability to reach a firm conclusion.^15^ In the past 2 years, studies have been published on some new RCTs that focused on acupuncture for CP/CPPS. Therefore, an overall systematic review should be conducted. We performed this systematic review to re-evaluate the efficacy and safety of acupuncture for treating CP/CPPS.

## MATERIALS AND METHODS

This systematic review was performed in accordance with the Preferred Reporting Items for Systematic Review and Meta-Analysis (PRISMA) statement,^16^ and the protocol of this systematic review and meta-analysis has been registered on PROSPERO (http://www.crd.york.ac.uk/PROSPERO). The registration number is CRD42015027522.

### Eligibility Criteria

#### Types of Studies

Only randomized controlled trials (RCTs) that evaluated the effects and/or safety data on acupuncture for CP/CPPS were included. For a trial to be included, it needed to contain adequacy randomization methods, eligibility diagnoses, eligibility outcome reports, and a description of statistical methods. The quality of studies was evaluated by professional assessors. We excluded articles focused on mechanisms and those without full text, animal trials, or reviews. There were no restrictions on language, publication date, or publication status.

#### Types of Participants

Participants diagnosed with CP/CPPS (category III as classified by the NIH) were considered. CP/CPPS was defined as urogenital pain, lower urinary tract symptoms with or without psychological issues, and sexual dysfunction over at least 3 out of the past 6 months in the absence of any urinary tract infection. Participants with acute bacterial prostatitis, a benign enlargement, prostate cancer, or other prostate diseases were excluded.

#### Types of Intervention

The following eligible comparisons were included: (a) acupuncture compared to Western drugs, (b) acupuncture supplementing Western drugs compared to the same Western drugs, and (c) acupuncture compared to sham/placebo acupuncture or a waiting group. Moreover, to meet the objective of this review, we focused on acupuncture that could be administered in a primary care setting, which included any type of penetrating acupuncture (i.e., acupuncture, electro-acupuncture, warm acupuncture, abdominal acupuncture, auricular acupuncture, etc.). We excluded trials evaluating the effectiveness of acupoints injection, needle-knife, and Chinese herbs, or acupuncture as a complement to the above interventions. Trials in which acupuncture was an adjunctive treatment combined with conventional Western medicine were included.

#### Types of Outcome Measures

The primary outcome was the changes in the total NIH-CPSI score after treatment.^17,18^

Secondary outcomes included changes in NIH-CPSI subscales, IPSS, and the global response rate. In addition, adverse events from interventions were recorded.

#### Data Sources and Searches

We used the review methods recommended in the handbook of the Cochrane Back Review Group. The following databases were searched from their inception until 30 November 2015: MEDLINE, EMBASE, CENTRAL, Web of Science, CBM, CNKI, Wang-Fang Database, JCRM, and CiNii. We also searched grey literature via Google Scholar and websites of the National Institute of Diabetes and Digestive and Kidney Diseases. The search terms related to acupuncture, chronic prostatitis, chronic pelvic pain syndrome, and randomized controlled trials. No language barriers were imposed.

(See Table 1, Supplemental Content which represents the strategy for searching in the PUBMED database).

### Study Selection

Two reviewers (JW and JZ) scanned the titles and abstracts independently and according to the eligibility assessments, which is protocol for systematic reviews. The search results will be imported to EndNote X7 software (Thomson Reuters, New York, NY) if available. Furthermore, duplications will also be eliminated by EndNote X7 software. Any disagreements between reviewers were resolved through discussion; if no agreement could be reached, a third rater would make a decision (ZL).

### Data Collection Process

Two independent reviewers (JW and JZ) extracted sample sizes, means, standard deviations, and percentages. For the purpose of this review, we extracted the change score of means and standard deviation, and when there was insufficient data in trial reports, we attempted to contact authors once a week (at least 3 times). If no one responded, we estimated data using the methods recommended in the Cochrane Handbook for Systematic Reviews of Interventions and using an imputed correlation coefficient of 0.5 (*R* = 0.5): 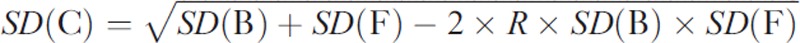


C, B, and F denote change, baseline, and final, respectively.^19^

We created a spreadsheet of data extraction according to Cochrane Consumers and Communication Review Group's data extraction template. A pilot collection (2 reviewers extracted the data of 3 randomized controlled trials independently, and once the procedure ended, data were checked by a third rater) was performed to examine and test the rule of collection. Any disagreements between reviewers were resolved through discussion; if no agreement could be reached, a third rater would make a decision (ZL).

### Data Items

The following data were extracted from each included trial: (a) characteristics of participants (nation, age, diagnosis); (b) types of treatments and control groups (for acupuncture, the acupuncture type, manipulation, duration, and frequency were recorded; for drugs, the type, dose, duration and frequency were noted); (c) outcome measures (the variety of scales as well as the global response rates); and (d) the follow-up period, if available.

### Risk of Bias Assessment

We used the Cochrane Collaboration tool to evaluate the risk of bias for the RCTs included.^20^ Two reviewers (ZQ and JW) independently evaluated the adequacy of random sequence generation and allocation concealment, blinding of participants, outcome assessors, incomplete outcome data, selective reporting, and other biases.^21^ Any disagreements were discussed and resolved by a third author (ZL).

To assess the possibility of publication bias, a funnel plot of the included trials is generally produced, where asymmetry of the funnel plot means that unpublished, smaller trials with negative results may be affecting the results of the meta-analysis. However, in this review, the low number of trials prevented use of a funnel plot and assessment of publication bias.

### Statistical Analysis

The primary outcome (total NIH-CPSI score) was calculated by computing the mean difference (MD) with 95% confidence intervals (CI). For secondary outcomes, data on continuous variables (i.e., NIH-CPSI subscores or IPSS) were calculated by computing MD with 95% CI. Data on dichotomous variables (i.e., the global response rate) were measured using the risk ratio (RR) with 95% CI. We used Revman V.5.3.3 software to synthesize extracted data and conduct a meta-analysis. The included trials administered different types of acupuncture, which can potentially cause heterogeneity of the results. Thus, a random effect model was used during meta-analysis. The presence of heterogeneity was assessed with Cochrane's *Q*-test and quantified with the *I*^2^ test. We accepted the potential heterogeneity when the *P* value was >0.1 in the *X*^2^ test and the *I*^2^ was <50%. In the case of heterogeneity, we attempted to identify and explore the heterogeneity using sensitivity analysis or subgroup analysis—if the quantity of included trials was available.

## RESULTS

### Study Selection

A total of 1054 studies were searched, in which 372 duplicates were omitted and the titles and abstracts of 682 studies remained to be reviewed. The remaining studies were excluded for the following reasons: 192 were non-RCTs, 57 were the experiences of experts, 84 were animal trials or mechanisms, and 128 did not meet our criteria of intervention. After scanning titles and abstracts, the full texts of the remaining 221 studies were examined in more detail. It appeared that 52 studies were excluded because they were not true RCTs, 105 studies were excluded because they included inappropriate interventions, 35 studies did not have clear diagnoses, which may have included participants with bacteria prostatitis or other prostate diseases. Twenty-two did not provide standard outcome measures. Therefore, a total of 7 studies were available for quantity and quality analysis in this review.^22–28^

**FIGURE 1:**
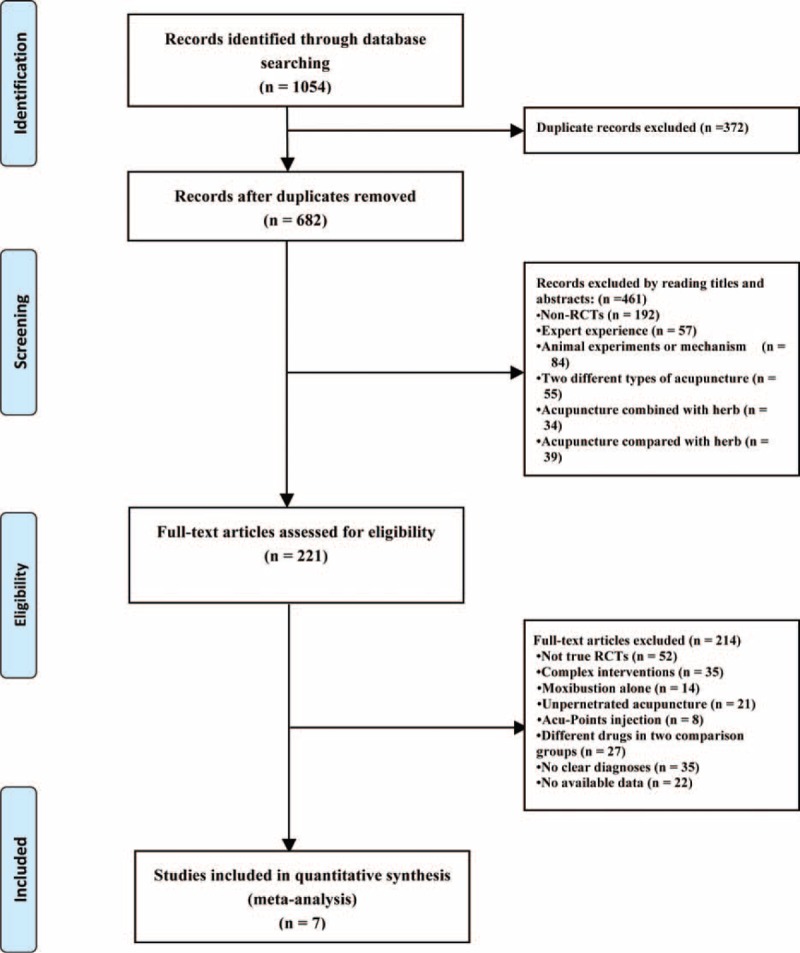
Flowchart of trial selection process for this systematic review.

See flow diagram, Figure 1.

### Study Description

Four trials were from Malaysia, Korea, and Turkey and were published in English,^22–24,27^ and 3 trials were from China and were published in Chinese.^25,26,28^ All 7 trials were single-centre, randomized controlled trials. Treatment sessions were from 4 weeks to 10 weeks. Three trials set the follow-up period from 18 weeks to 24 weeks.^22,24,27^

#### Participants

The included studies involved at total of 471 adult male participants, and 399 participants were included in meta-analysis.^22–28^ Three trials involving 231 participants indicated they only included type IIIB,^22,27,28^ and participants of type IIIA were excluded; another 4 trials involving 240 participants included III types whether they were A or B.

#### Intervention

Two trials used electro-acupuncture (EA) as an intervention.^22,23^ Five trials used manual acupuncture (MA) as an intervention, in which 1 trial used MA plus medicine (levofloxacin) as an intervention.^26^ Based on the prior protocol we followed, we included trials comparing acupuncture with sham/placebo acupuncture, no treatment, or conventional Western drugs as the control group. Four trials used drugs in the control procedure.^22,25,26,28^ Four trials used penetrating acupuncture on nonacupuncture points.^23,24,27,28^

#### Outcomes

In all studies the primary outcome assessed was the difference in total NIH-CPSI score between baseline and study completion. Most trials did not provide the variety of decreasing scores but rather the data on baseline and endpoints—with the exception of 2 trials.^22,24^ Based on our protocol, we took advantage of the methods recommended in the Cochrane Handbook to calculate and evaluate the changed data from the data they provided. In addition, the global response rate, NIH-CPSI subscale score, and IPSS were provided as the secondary outcomes of some trials.^22–24,26-28^

(see Table 2 and 3, Supplemental Content which summarizes the parameters of included trials).

### Risk of Bias Within Studies

Two reviewers used the risk of bias (ROB) tool recommended by the Cochrane Handbook to evaluate included trials, and there was good agreement between reviewers. Not all RCTs provided the information on subsequent allocation; thus, all 7 trials were rated as having unclear risk of bias in this domain. Three RCTs comparing acupuncture to medicine did not provide adequate blinding information, and we believe this methodological limitation may potentially affect the findings. As a result, these 3 trials were rated as having a high risk of bias in this domain.^22,26,28^ Two RCTs provided the total NIH-CPSI score without subscores and did not report adverse events or drop offs; we thus figured there was a risk of incomplete outcome data and selective outcome reporting between the 2 trials.^25,26^ One RCT with a small group size (n = 36, 3 groups) was rated as high risk in other sources of bias domain.^23^ (See Table 4 Supplemental Content, which represents the ROB of included RCTs).

### Acupuncture Versus Sham Acupuncture

**FIGURE 2:**
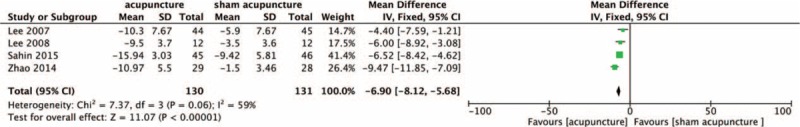
Forest plot of the effect of acupuncture versus sham acupuncture on the NIH-CPSI total score using the random model. NIH-CPSI = National Institute of Health Chronic Prostatitis Symptom Index.

**FIGURE 3:**
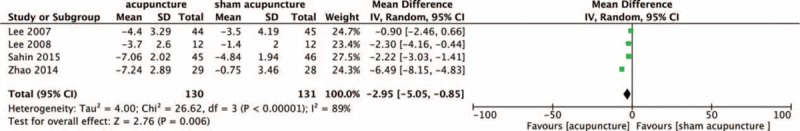
Forest plot of the effect of acupuncture versus sham acupuncture on the NIH-CPSI pain domain score using the random model. NIH-CPSI = National Institute of Health Chronic Prostatitis Symptom Index.

**FIGURE 4:**
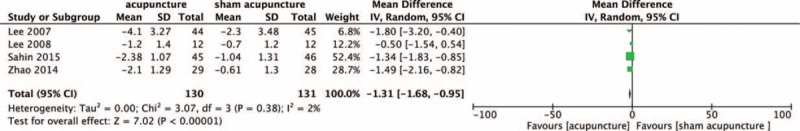
Forest plot of the effect of acupuncture versus sham acupuncture on the NIH-CPSI voiding domain score using the random model. NIH-CPSI = National Institute of Health Chronic Prostatitis Symptom Index.

**FIGURE 5:**
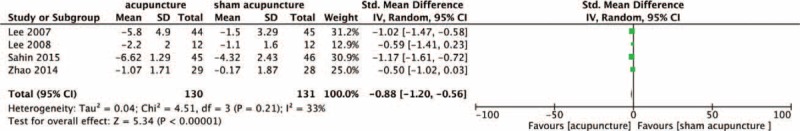
Forest plot of the effect of acupuncture versus sham acupuncture on the NIH-CPSI Qof domain score using the random model. NIH-CPSI = National Institute of Health Chronic Prostatitis Symptom Index, Qof = quality of life.

**FIGURE 6:**
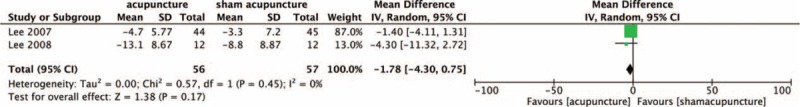
Forest plot of the effect of acupuncture versus sham acupuncture on the IPSS score.

**FIGURE 7:**
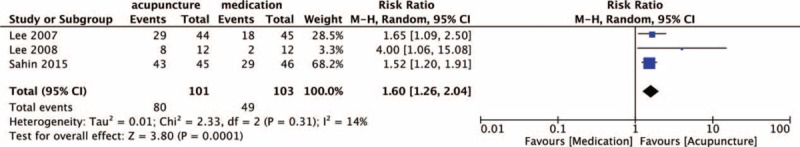
Forest plot of the effect of acupuncture versus sham acupuncture on the global assessment.

**FIGURE 8:**
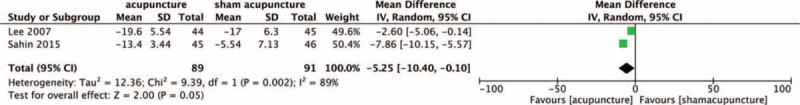
Forest plot of the effect of acupuncture versus sham acupuncture on the NIH-CPSI total score in follow-up. NIH-CPSI = National Institute of Health Chronic Prostatitis Symptom Index.

1. NIH-CPSI total score: Sham acupuncture involves choosing nonacupoints (superficial and/or 10–15 mm to the left of each correct acupoint). Four RCTs involving 261 participants comparing acupuncture to sham acupuncture reported changes in the total NIH-CPSI score. Meta-analysis of 4 trials yielded a significant difference in favor of acupuncture (MD: −6.09 [95%CI: −8.12 to −5.68]) with moderate heterogeneity (*I*^2^ = 59%).^23,24,27,28^ (Figure 2)
2. NIH-CPSI pain domain subscore: 4 RCTs involving 261 participants compared acupuncture to sham acupuncture. In the pain domain score, meta-analysis showed an average pain score reduction of 2.95 points (MD: −2.95 [95%CI: −5.05 to −0.85]) with high heterogeneity (*I*^2^ = 89%) (Figure 3). A sensitivity analysis succeeded in identifying the source of heterogeneity: 1 trial conducted by Zhao and Sun. After eliminating this trial from data combining, the heterogeneity vanished and could be accepted (*I*^2^ = 12%) with an average pain reduction of 1.95 (MD: −1.95 [95%CI: −2.71 to −1.19]).^23,24,27,28^ (See Figure 1 Supplemental Content, which illustrates the sensitivity analysis.)
3. NIH-CPSI voiding domain subscore: 4 RCTs involving 261 participants that compared acupuncture to sham acupuncture found an average reduction of 1.31 points in the NIH-CPSI voiding score. Meta-analysis showed a significantly greater improvement for acupuncture over sham acupuncture (MD: −1.31 [95%CI: −1.68 to −0.95]) with low heterogeneity (*I*^2^ = 2%)^23,24,27,28^ (Figure 4).
4. NIH-CPSI quality of life domain subscore: for improvement in quality of life, the result of meta-analysis of 4 trials involving 261 participants indicated that compared with sham acupuncture, real acupuncture could improve the quality of life in patients with CP/CPPS (MD: −0.88 [95%CI: −1.20 to −0.56]) with moderated heterogeneity (*I*^2^ = 33%)^23,24,27,28^ (Figure 5).
5. IPSS score: 2 trials involving 113 participants evaluated total IPSS score as a secondary outcome. In the results of meta-analysis, no significant differences were found among the treatment group (MD: −1.78 [95%CI: −4.30 to 0.75]) with *I*^2^ = 0%^23,24^ (Figure 6).
6. Global response rate: 3 trials involving 129 participants reported global assessment as one of the outcomes. According to these trials, a participant who has a more than a 6-point decrease in total NIH-CPSI score after treatment can be considered a responder. A meta-analysis of the data showed favorable effects of acupuncture on improving the global assessment (RR: 1.60 [95%CI: 1.26 to 2.04]) with low heterogeneity (*I*^2^ = 14%)^23,24,27^ (Figure 7).
7. Long-term follow-up response: 2 trials involving 180 participants observed long-term effects by measuring the changes in the total NIH-CPSI score after follow-up. One of the trials reported the NIH-CPSI subscale score after follow-up as well. After combining data, the result showed a significant difference in favor of acupuncture over sham acupuncture after 20 more weeks of follow-up (MD: −5.25 [95%CI: −10.40 to −0.1]) with a high heterogeneity of *I*^2^ = 89%. Because of the insufficient studies included, subgroup analyses or sensitivity analyses failed to explore the source of heterogeneity. As a result, the evidence of combing data has been limited. The source of heterogeneity may relate to different follow-up periods and the number of acupoints selected^24,27^ (Figure 8).

### Acupuncture Versus Medication

**FIGURE 9:**
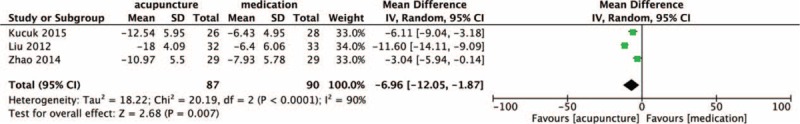
Forest plot of the effect of acupuncture versus medication on the NIH-CPSI total score. NIH-CPSI = National Institute of Health Chronic Prostatitis Symptom Index.

**FIGURE 10:**
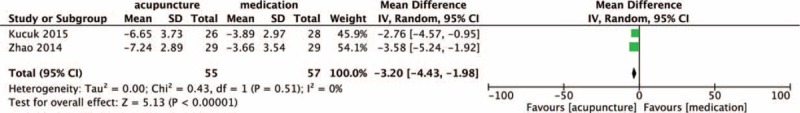
Forest plot of the effect of acupuncture versus medication on the NIH-CPSI pain domain score. NIH-CPSI = National Institute of Health Chronic Prostatitis Symptom Index.

**FIGURE 11:**
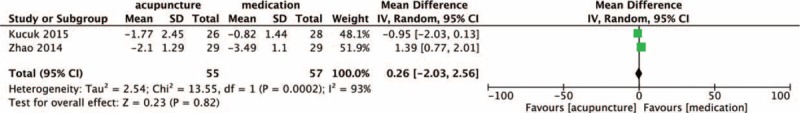
Forest plot of the effect of acupuncture versus medication on the NIH-CPSI voiding domain score. NIH-CPSI = National Institute of Health Chronic Prostatitis Symptom Index.

**FIGURE 12:**
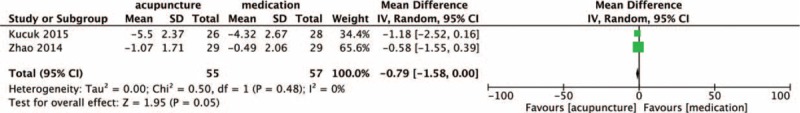
Forest plot of the effect of acupuncture versus medication on the NIH-CPSI Qof domain score. NIH-CPSI = National Institute of Health Chronic Prostatitis Symptom Index, Qof = quality of life.

**FIGURE 13:**
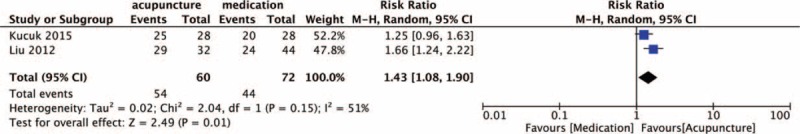
Forest plot of the effect of acupuncture versus medication on the global assessment.

1. NIH-CPSI total score: 3 trials involving 177 participants that compared acupuncture to medication (Alfuzosin, Tamsulosin, and Prostat) reported the total NIH-CPSI score as the primary outcome and found an average reduction of 6.96 points (MD: −6.96 [95%CI: −12.05 to −1.87]) with high heterogeneity (*I*^2^ = 90%)^22,25,28^ (Figure 9). To identify the source of heterogeneity, sensitivity analyses were conducted. We determined the source of heterogeneity to be the RCT conducted by Liu et al.^25^ The unique form of acupuncture manipulation administered in this study may be the cause of the notably positive outcome. The trial used a sort of acupuncture manipulation called “Shuang Gu Yi Tong” and selected acupoints distributed on the head. After eliminating this study the *I*^2^ value decreased to 53% with the combined effect size (MD: −4.57 [95%CI: −7.58 to −1.56]).^22,28^ (See Figure 2 Supplemental Content, which illustrates the sensitivity analysis.)
2. NIH-CPSI pain domain subscore: 2 trials involving 112 participants compared acupuncture to medicine (Levofloxacinand and Tamsulosin) in terms of pain symptoms by measuring the NIH-CPSI pain domain subscore. The result of meta-analysis indicated that acupuncture is more effective than medication (MD: −3.20 [95%CI: −4.43 to −1.98]) with *I*^2^ = 0%^22,28^ (Figure 10).
3. NIH-CPSI voiding domain subscore: 2 trials involving 112 participants compared acupuncture to medication (Levofloxacinand and Tamsulosin). The result was that acupuncture is not significantly different from medication (MD: 0.26 [95%CI: −2.03 to 2.56]) with high heterogeneity (*I*^2^ = 93%). Because there were insufficient RCTs included, subgroup analyses and sensitivity analyses failed to identify the source of heterogeneity^22,28^ (Figure 11).
4. NIH-CPSI quality of life domain subscore: 2 trials involving 112 participants compared acupuncture to medication (Levofloxacinand and Tamsulosin). The results of meta-analysis showed that acupuncture did not have a significant effect (MD: −0.79 [95%CI: −1.58 to 0.00]) with an *I*^2^ value of 0%^22,28^ (Figure 12).
5. Global assessment: 2 trials involving 98 participants evaluated acupuncture and medicine (Levofloxacinand and Prostat) for a global assessment. After combing data, the result indicated that patients treated with medication failed to report an improvement in their symptoms more frequently than patients treated with acupuncture (RR: 1.43 [95%CI: 1.08–1.90]) with moderate heterogeneity (*I*^2^ = 51%)^22,25^ (Figure 13).

### Acupuncture Plus Medication Versus the Same Medication

NIH-CPSI total score and subscores: a single trial published in Chinese and including 60 participants was rated as having a high risk of bias; it compared acupuncture plus terazosin hydrochloride to administering terazosin hydrochloride alone.^26^ The use of Terazosin in the 2 groups was the same. The result showed that compared to terazosin alone, acupuncture plus terazosin had a greater effect on decreasing the total NIH-CPSI score (MD: −4.40 [95%CI: −6.85 to −1.95]). Meanwhile, it concluded that patients who received acupuncture plus terazosin experienced significant pain relief (MD: −2.66 [95%CI: −4.19 to −1.13]) and quality of life improvement (MD: −1.87 [95%CI: −2.78 to −0.96]). The urinary symptom score was negative (MD: −0.13 [95%CI: −1.02 to 1.28]).

### Adverse Events

One of the 7 trials reported on the occurrence of adverse events (ADs) in the acupuncture group;^24^ 2 trials reported no ADs,^22,27^ and other trials did not provide information related to ADs.^25,26,28^ Overall, 1 trial calculated for ADs in all (6 hematomas, 2 with pain at needling sites).^24,32^

## DISCUSSION

### Summary of Findings

This systematic review was conducted to provide an update to prior reviews of acupuncture for CP/CPPS. So far, acupuncture has been used for management of symptoms in patients with CP/CPPS in many countries. Although the reported data came from uncontrolled series trials, the results suggested that acupuncture might provide benefits for relieving the symptoms of this syndrome.^29–32^ Due to the poorly understood etiology and pathology of this syndrome, symptom control is increasingly recognized as an important goal for patients with CP/CPPS, according to a systematic review aiming to assess all trials reporting on therapeutic intervention for CP/CPPS using the NIH-CPSI.^21^ Results showed that many current treatments for CP/CPPS are largely ineffective, including alpha-blockers and antibiotics. Acupuncture was found to produce statistically and clinically significant reductions in the NIH-CPSI voiding domain, but due to the insufficient number of trials, further research is needed to explore the use of acupuncture for CP/CPPS.^33^ In this review, we included RCTs comparing acupuncture to sham acupuncture or to conventional Western medicine and conducted meta-analysis to assess the effectiveness and safety of acupuncture for CP/CPPS.

On the basis of our analysis of 7 RCTs, we found that the current level of evidence supports the benefits of using acupuncture to improve symptoms in CP/CPPS patients, especially in the pain relief domain. Four trials examined acupuncture compared to sham acupuncture in terms of decreasing NIH-CPSI and reported positive outcomes; 2 of them were rated as having unclear risk of bias,^24,27^ whereas the other 2 trials were rated as high.^23,25^ To explore the efficacy of acupuncture, we combined the data among the trials and calculated the mean difference by comparing the outcomes of the sham acupuncture arm between baseline and endpoint after treatment. According to a consensus of expert opinion and data from a multicentre trial, a 6-point decrease in the total NIH-CPSI score between study completion and baseline can be seen as a minimum clinically important difference (MCID).^10^ The result of the meta-analysis showed the MD of change in total NIH-CPSI score is −6.09. This suggests that there are specific effects associated with acupuncture, at least over the short term. Two trials compared acupuncture to sham acupuncture, and the result of combing data was observed with high heterogeneity, which indicated that acupuncture may have long-term effects, but the evidence was limited. The between-group effect size estimates were insignificant among several trials, which suggested that nonspecific factors might have an impact on the treatment, yet the specific effects are greater. Another result of the meta-analysis, including 2 trials that focused on IPSS, conflicted with previous results by demonstrating that sham acupuncture was as effective as acupuncture in controlling urinary symptoms (as observed in the IPSS score).^23,24^ The contradicting sources are difficult to determine because neither subgroup analysis nor sensitivity analysis can be conducted with no other trials providing available information. We list our own theories herein. First, there was a different set of questions designed in the 2 questionnaires, despite the fact that 2 questionnaires can be used to quantitatively evaluate the severity of symptoms and both of them are excellent choices for evaluating the effectiveness of treatments. However, NIH-CPSI aimed to evaluate symptoms of CP/CPPS, and IPSS was designed for benign prostatic hypertrophy. We estimate that the design of the scales themselves might lead to this diversity. Second, the inadequate number of RCTs included, as well as the insufficient sample size, might also explain the conflicting results. Further studies are needed to explore the different evaluation effects between the NIH-CPSI voiding domain subscale and the IPSS scale.

In pharmaceutical controlled research, due to insufficient RCTs included to compare acupuncture to medicine and also because each trial administered a different sort of medicine (NSAIDs, alpha-blockers or antibiotics), the results of data combing showed significant heterogeneity, which makes the interpretation of findings difficult. Results showed that compared to medicine, acupuncture leads to a significant decrease in the total NIH-CPSI score and pain symptom subscale score; in terms of urinary symptoms and quality of life improvement, acupuncture was as effective as medicine. Because the included trials involving medicine as a control treatment were insufficient, it is difficult for investigators to perform subgroup analysis or sensitivity analysis. The heterogeneity in meta-analysis of voiding symptoms was observed to be high, and we believe the explanation is the different medication administered (including usage, dose, and treatment sessions) in each trial. One trial published in Chinese reported positive outcomes but was rated as having unclear risk of bias; it found that acupuncture plus medicine (terazosin hydrochloride) is superior to that particular medicine alone. This trial emphasizes that complementary use of acupuncture for symptom control in CP/CPPS is important to consider.

There are several possible interpretations of these findings caused by a mixture of different types of interventions and treatment sessions. Compared with medicine, acupuncture is effective and safe for patients with CP/CPPS in terms of decreasing the total NIH-CPSI score, particularly in pain relief. With regard to the voiding domain and quality of life domain, acupuncture is more effective than sham acupuncture (inserting a needle into sham acupoints) and may be as effective as medicine. In addition, acupuncture may be more effective as a complement to medicine than medication alone.

### Comparisons With Previous Study

In 2012, a systematic review that included 9 Asian RCTs was published and concluded that acupuncture is an encouraging therapy for CP/CPPS.^15^ We sought to identify all relevant trials in the previous review. Given our rigorous criteria, most of the included trials published in Chinese were unavailable for data abstraction, as they did not provide adequate information related to the characteristics of participants as well as outcome measures. Posadzki et al noted that the quantity and the quality of existing evidence is also insufficient to draw firm conclusions. Moreover, this review included global assessment as an outcome measure. However, as mentioned above, most relevant trials included in this review did not describe detailed information about outcomes. As a result, conclusions are limited. On the other hand, due to the quantity and quality of the included trials, the authors of the previous systematic review did not conduct a meta-analysis of NIH-CPSI total score, nor did they do so for subscale score or any other measures. In terms of interventions, it included insufficient trials comparing acupuncture with sham acupuncture. Thus, it is difficult to combine the data. Furthermore, no follow-up data has been collected for the previous systematic review. Hence, evaluation of the long-term effects of acupuncture was unsuccessful. In this systematic review, we formulated rigorous standards for including RCTs to guarantee the quality of evidence. We also extracted data from graded uniform outcome measurements that have been widely accepted (NIH-CPSI) to provide generalized evidence using quantity analysis.

### Limitations

This review has several limitations that should be addressed. First, although every study provided before-and-after treatment data, only 2 of them had the change in value as a primary outcome. Therefore, to calculate the difference of mean as well as the standard deviation, we estimated the missing data by assuming the correlation coefficient *R* was 0.5, a conservative value that leads to the highest variance. Second, the mixture of different types of acupuncture, frequency of administration, duration of each session, and location of acupoints may have a potential impact on the effects of acupuncture. However, because the included trials were insufficient, it is difficult to conduct subgroup analysis or meta-regression to avoid this methodological limitation. All of the trials lacked the details of concealment and most of them did not provide adequate information on blinding either. Because of the characteristic of acupuncture, it is difficult to conduct blinding in patients, especially the trial that included a control group with drugs administered. However, for acupuncture, blinding to assessors is one of the cardinal methods to enable the generalizability of findings. Moreover, due to the lack of reporting on placebo-controlled trials that compare acupuncture to nonpenetrated acupuncture, placebo effects are impossible to eliminate. The specific effects of acupuncture needling are not well understood.

## CONCLUSION

In conclusion, the evidence supported acupuncture as an effective treatment to improve symptoms of CP/CPPS. Compared with sham acupuncture, real acupuncture leads to significant reductions in the pain, urinary symptoms, and quality of life domains of the NIH-CPSI. Compared with conventional Western medicine, acupuncture may be more effective in decreasing the total NIH-CPSI score, especially in terms of pain relief. With regard to urinary symptoms and quality of life, there is no significant difference between acupuncture and conventional Western medicine. Current existing evidence allows limited conclusions to be reached through comparing acupuncture and medicine, and additional trials are needed to improve the reliability of these findings. In terms of adverse events, acupuncture was linked to rare and slightly adverse events such as hematomas or pain, but these resolved quickly and no other serious events have been reported. In addition, the use of acupuncture as an appropriate adjunctive treatment for symptom control in patients with CP/CPPS should be considered.

## Supplementary Material

Supplemental Digital Content
