# Supplementary material for: Systematic Review of Acupuncture for Chronic Prostatitis/Chronic Pelvic Pain Syndrome
Source: Medicine (Baltimore). 2016 Mar 18;95(11):e3095. doi: 10.1097/MD.0000000000003095 (PMC4839929; doi:10.1097/MD.0000000000003095)
Supplement: Supplemental Digital Content [file medi-95-e3095-s001.pdf]

	acupuncture			sham acupuncture				Mean Difference
Study or Subgroup	Mean	SD	Total	Mean	SD	Total	Weight	IV, Random, 95% CI
Lee 2007	−4.4	3.29	44	−3.5	4.19	45	21.5%	−0.90 [−2.46, 0.66]
Lee 2008	−3.7	2.6	12	−1.4	2	12	15.7%	−2.30 [−4.16, −0.44]
Sahin 2015	−7.06	2.02	45	−4.84	1.94	46	62.8%	−2.22 [−3.03, −1.41]
**Total (95% CI)**			**101**			**103**	**100.0%**	**−1.95 [−2.71, −1.19]**

Heterogeneity: $Tau^2 = 0.07$; $Chi^2 = 2.28$, $df = 2$ ($P = 0.32$); $I^2 = 12\%$
Test for overall effect: $Z = 5.01$ ($P < 0.00001$)

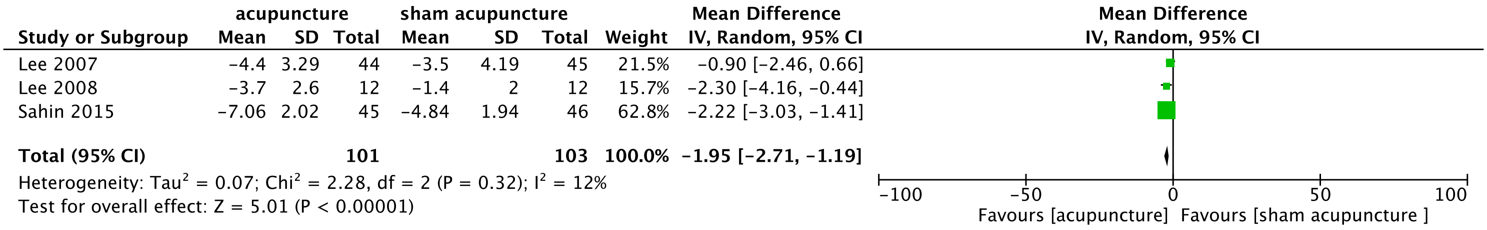

Study or Subgroup	acupuncture Mean	acupuncture SD	acupuncture Total	medication Mean	medication SD	medication Total	Weight	Mean Difference IV, Random, 95% CI	Mean Difference IV, Random, 95% CI
Eyup 2014	−12.54	5.95	26	−6.43	4.95	28	49.8%	−6.11 [−9.04, −3.18]	
Zhao 2014	−10.97	5.5	29	−7.93	5.78	29	50.2%	−3.04 [−5.94, −0.14]	
**Total (95% CI)**			**55**			**57**	**100.0%**	**−4.57 [−7.58, −1.56]**	

Heterogeneity: $Tau^2 = 2.50$; $Chi^2 = 2.13$, df = 1 (P = 0.14); $I^2 = 53\%$
Test for overall effect: Z = 2.98 (P = 0.003)

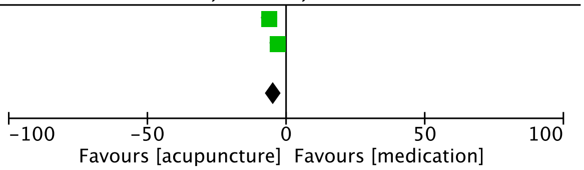

Supplemental Table 1 Search strategy used in PubMed database

No.	Search Items
1	randomized controlled trial.pt
2	controlled clinical trial.pt
3.	randomized.ti,ab
4.	randomly.ti,ab
5.	groups.ti,ab
6.	trial.ti,ab
7.	or 1-6
8.	acupuncture.ti,ab
9.	electro-acupuncture.ti,ab
10.	elongated needle.ti,ab
11.	three edged needle.ti,ab
12.	(fire needle or warming needle).ti,ab
13.	auricular acupuncture.ti,ab
14.	abdominal acupuncture.ti,ab
15.	warm acupuncture.ti,ab
16.	pyonex.ti,ab
17.	or 8-16
18.	chronic prostatitis.ti,ab
19.	chronic pelvic pain syndrome.ti,ab
20.	non-bacterial prostatitis.ti,ab
21.	prostatitides.ti,ab
22.	inflammatory prostatitides.ti,ab
23.	inflammatory prostatitis.ti,ab
24.	asymptomatic inflammatory prostatitis.ti,ab
25.	or 18-24
26.	7 and 17 and 25

This search strategy will be modified as required for other electronic databases.

Supplemental Table 2 Characteristics of included randomized controlled trials

Study	Location (published language)	Sample size	Diagnosis	Interventions of treatment group	Intervention of control group	Test duration (week)	Outcomes	Adverse event
**Sham controlled**								
**Lee et al 2008**[24]	Malasia (English)	89 (44/45)	CP/CPPS	Needle parameter: 0.3mm stainless needles<br>Depth: 40-60mm, depending on location of acupoints<br>Points: CV1, CV4, SP6, SP9<br>Duration: 30mins, twice a week<br>Manipulation: rotation	Needle parameter: 0.3mm stainless needles<br>Depth: 25mm for all points<br>Points: 15mm to the left of actual CV1, CV4, SP6, SP9<br>Duration: 30mins, twice a week<br>Manipulation: manual acupuncture	10	NIH-CPSI<br>IPSS<br>IIEF<br>Global assessment	8 in the acupuncture group (6 hematoma and 2 with pain at needling sites)<br>5 in the sham group (1 hematoma, 3 with pain at needling sites and 1 with acute urinary retention)
**Lee et al 2009**[23]	Korea (English)	36 (12/12/12)	CP/CPPS	Needle parameter: 0.25mm stainless needles<br>Depth: 30mm<br>Points: BL32, BL33, GB30<br>Duration: 20mins, twice a week<br>Manipulation: The needles were stimulated electrically with a frequency of 4Hz and an intensity of 5-10mA with continuous stimulation.	Needle parameter: 0.25mm stainless needles<br>Depth: 30mm<br>Points: 15 mm to the lateral of BL32, BL33, GB30<br>Duration: 20mins, twice a week<br>Manipulation: None	6	NIH-CPSI<br>IPSS<br>Global assessment	1 in the sham group (lower back pain near the needling site that resolved quickly)
**Sahin et al 2015**[27]	Turkey (English)	91 (45/46)	CP/CPPS IIIB	Needle parameter: 0.3mm stainless needles<br>Depth: 25-30mm<br>Points: BL33, BL34, BL54,CV1,CV4,SP6,SP9<br>Duration: 20mins, once a week<br>Manipulation:rotation	Needle parameter: 0.3mm stainless needles<br>Depth: 25-30mm<br>Points: 10 mm left of BL33, BL34, BL54,CV1,CV4,SP6,SP9<br>Duration: 20mins, once a week<br>Manipulation:rotation	6	NIH-CPSI	No adverse events were reported in both groups
**Zhao et al 2014**[28]	China (Chinese)	86 (29/29/28)	CP/CPPS IIIB	Needle parameter: NR<br>Depth: NR<br>Points: LU7, SI3, SP4<br>Duration: 20mins, twice a week<br>Manipulation:rotation	Needle parameter: NR<br>Depth: NR<br>Points: 15 mm to the lateral of LU7, SI3, SP4<br>Duration: 20mins, twice a week<br>Manipulation: rotation	4	NIH-CPSI	NR
**Medicine controlled**								
**Kucuk et al 2015**[22]	Turkey (English)	54 (26/28)	CP/CPPS IIIB	Needle parameter: 0.25mm stainless needles<br>Depth: 25-30mm<br>Points: UP28, GB41, LIV3, SP6, SP8, LI4<br>Duration: 20mins, twice a week<br>Manipulation: electro-acupuncture	levofloxacin 500 mg daily and ibuprofen 200 mg twice a day for 6 weeks	7	NIH-CPSI	No adverse events were reported in both groups
**Zhao et al 2014**[28]	China (Chinese)	86 (29/29/28)	CP/CPPS IIIB	Needle parameter: NR<br>Depth: NR<br>Points: LU7, SI3, SP4<br>Duration: 20mins, twice a week<br>Manipulation:rotation	Tamsulosin 0.2 mg daily for 4 weeks	4	NIH-CPSI	NR
**Liu et al 2012**[25]	China (Chinese)	55 (32/33)	CP/CPPS	Needle parameter: 0.3mm stainless needles<br>Depth: 100mm for CV54, 30mm for others<br>Points: CV54,CV4, ST28, SP6, LIV3, EX-HN1<br>Duration: 20mins, three times a week<br>Manipulation:rotation	Prostat 70 mg twice a day and fluoxetine 20 mg daily for 4 weeks	4	NIH-CPSI<br>Global assessment	NR
**Qi et al 2012**[26]	China (Chinese)	60 (30/30)	CP/CPPS	Needle parameter: NR<br>Depth: 25-50mm<br>Points: CV1,CV3, CV4, SP9, SP10<br>Duration: 30mins, twice a week<br>Manipulation:rotation<br>Terazosin 2mg daily for 4 weeks	Terazosin 2mg daily for 4 weeks	4	NIH-CPSI	NR

NIH-CPSI: National Institute of Health Chronic Prostatitis Symptom Index; IPSS: International Prostate Symptom Score; IIEF: International Index of Erectile Function; NR: no report

Supplental Table 3 NIH-CPSI outcomes of randomized controlled trials

Study	Group	Participants	NIH-CPSI							
			Baseline / After treatment (mean±SD)				Changes (mean±SD)			
			Total score	Pain domain	Voiding domain	Qof domain	Total score	Pain domain	Voiding domain	Qof domain
**Lee et al 2008[24]**	Acu	44	24.8±6.2 / 14.5±8.7	11.4±3.4 / 7±4.5	4.1±3.7 / 0±2.9	9.1±1.6 / 3.5±5.5	10.3±7.7	4.4±3.3	4.1±3.3	5.8±4.9
	SAc	45	25.2±5.8 / 19±10.3	11.5±2.9 / 8±4.8	4.3±3.7 / 2±3.2	9.4±1.8 / 8±3.8	5.9±7.7	3.5±4.2	2.3±3.5	1.5±3.3
**Lee et al 2009[25]**	Acu	12	26.9±5.2 / NR	12.2±2.5 / NR	5.3±3 / NR	9.4±1.8 / NR	9.5±3.7	5.2±1.9	1.8±1.5	2.6±2.4
	SAc	12	25.5±3.6 / NR	11.8±2.7 / NR	3.8±2.8 / NR	9.8±1.6 / NR	3.5±3.6	1.6±1.8	0.7±1.4	1.3±1.8
**Sahin et al 2015[27]**	Acu	45	27±3.5 /10.8±1.9	13.2±2.3 / 6.1±1.5	4.6±1.2 / 2.2±0.9	9.1±1.5 / 2.5±0.8	15.9±3	7.1±2	2.4±1.1	6.6±1.3
	SAc	46	26.5±3.7 / 16.2±6.6	13±2.2 / 8.2±3.2	4.5±1.4 / 3.5±1.2	8.9±1.4 / 4.6±2.8	9.4±5.8	4.8±1.9	1±1.3	4.3±2.4
**Zhao et al 2014[28]**	Acu	29	24.5±6.3 / 13.5±3.6	13.7±3.2 / 6.5±2.4	5.4±1.5 / 3.3±1	5.3±1.9 / 4.2±1.4	10.9±5.5	7.2±2.9	2.1±1.3	1.1±1.7
	SAc	29	25.6±6.4 / 24±5.5	14.1±3.6 / 13.3±3.3	6±1.5 / 5.4±1.4	5.4±2 / 5.3±1.7	1.5±3.5	0.8±3.5	0.6±1.3	0.2±1.9
	Med	28	26.6±6.4 / 18.7±4.8	14.4±4 / 10.8±2.9	6.2±1.1 / 2.7±1.1	6±2.3 / 4.5±1.5	7.9±5.8	3.7±3.5	3.5±1.1	0.5±2.1
**Kucuk et al 2015[22]**	Acu	26	NR	NR	NR	NR	12.5±6	6.7±3.7	1.8±2.5	5.5±2.4
	Mem	28	NR	NR	NR	NR	6.4±5	3.9±3	0.8±1.4	4.3±2.7
**Liu et al 2012[25]**	Acu	32	27.9±4.7 / 9.9±2.7	NR	NR	NR	18±4.1	NR	NR	NR
	Med	33	25.8±5.9 / 19.4±6.2	NR	NR	NR	6.4±6.1	NR	NR	NR
**Qi et al 2012[26]**	Acu+Med	30	24.1±5.5 / 13.2±5.8	13.1±3.2 / 6.4±2.9	2.3±2.2 / 1.3±1.6	9±1.6 / 5.5±2.3	10.9±5.6	6.7±3	1±2	3.5±2
	Med	30	23.1±4.2 / 16.6±3.5	3±2.7 / 7.5±2.1	3±2.7 / 2.1±2.2	8.5±1.6 / 6.9±1.5	6.5±3.9	4.1±3	0.9±2.5	1.6±1.5

NIH-CPSI: National Institute of Health Chronic Prostatitis Symptom Index; Acu: acupuncture; SAc: sham acupuncture; Med: medication; NR: no report

Supplemental Table 4 ROB for included randomized controlled trials

Study	Sequence Generation	Allocation Concealment	Blinding	Incomplete Outcome Data	Selective Outcome Reporting	Other Sources of Bias
**Lee et al 2008[24]**	+	0	+	+	+	+
**Lee et al 2009[23]**	+	0	+	+	+	-
**Sahin et al 2015[27]**	+	0	+	+	+	+
**Zhao et al 2014[28]**	+	0	-	0	0	0
**Kucuk et al 2015[22]**	+	0	-	-	+	+
**Liu et al 2012[25]**	+	0	0	-	-	0
**Qi et al 2012[26]**	+	0	-	-	-	0

ROB: risk of bias; + indicates low risk of bias; 0, unclear; -, high risk of bias.
